# The Prognostic Significance of Histone Demethylase *UTX* in Esophageal Squamous Cell Carcinoma

**DOI:** 10.3390/ijms19010297

**Published:** 2018-01-19

**Authors:** Shau-Hsuan Li, Hung-I Lu, Wan-Ting Huang, Wan-Yu Tien, Ya-Chun Lan, Wei-Che Lin, Hsin-Ting Tsai, Chang-Han Chen

**Affiliations:** 1 Department of Hematology-Oncology, Kaohsiung Chang Gung Memorial Hospital and Chang Gung University College of Medicine, Kaohsiung 833, Taiwan; lee.a0928@msa.hinet.net (S.-H.L.); wanyu1001@gmail.com (W.-Y.T.); amylan1226@gmail.com (Y.-C.L.); 2 Department of Thoracic & Cardiovascular Surgery, Kaohsiung Chang Gung Memorial Hospital and Chang Gung University College of Medicine, Kaohsiung 833, Taiwan; luhungi@cgmh.org.tw; 3 Department of Pathology, Kaohsiung Chang Gung Memorial Hospital and Chang Gung University College of Medicine, Kaohsiung 833, Taiwan; Huangminnie@cgmh.org.tw; 4 Department of Diagnostic Radiology, Kaohsiung Chang Gung Memorial Hospital and Chang Gung University College of Medicine, Kaohsiung 833, Taiwan; alex@cgmh.org.tw; 5 Guangdong Institute of Gastroenterology, and Guangdong Provincial Key Laboratory of Colorectal and Pelvic Floor Disease, Sun Yat-sen University, Guangzhou 510020, China; thtsophia66@gmail.com; 6 Department of Applied Chemistry, and Graduate Institute of Biomedicine and Biomedical Technology, National Chi Nan University, Nantou 54561, Taiwan; 7 Center for Infectious Disease and Cancer Research, Kaohsiung Medical University, Kaohsiung 807, Taiwan

**Keywords:** esophageal cancer, squamous cell carcinoma, *UTX*, E-cadherin

## Abstract

The dysregulation of the ubiquitously transcribed TPR gene on the X chromosome (*UTX*) has been reported to be involved in the oncogenesis of several types of cancers. However, the expression and significance of *UTX* in esophageal squamous cell carcinoma (ESCC) remains largely undetermined. Immunohistochemistry was performed in 106 ESCC patients, and correlated with clinicopathological features and survival. The functional role of *UTX* in ESCC cells was determined by *UTX*-mediated siRNA. Univariate analyses showed that high *UTX* expression was associated with superior overall survival (OS, *p* = 0.011) and disease-free survival (DFS, *p* = 0.01). *UTX* overexpression was an independent prognosticator in multivariate analysis for OS (*p* = 0.013, hazard ratio = 1.996) and DFS (*p* = 0.009, hazard ratio = 1.972). The 5-year OS rates were 39% and 61% in patients with low expression and high expression of *UTX*, respectively. Inhibition of endogenous *UTX* in ESCC cells increased cell viability and BrdU incorporation, and decreased the expression of epithelial marker E-cadherin. Immunohistochemically, *UTX* expression was also positively correlated with E-cadherin expression. High *UTX* expression is independently associated with a better prognosis in patients with ESCC and downregulation of *UTX* increases ESCC cell growth and decreases E-cadherin expression. Our results suggest that *UTX* may be a novel therapeutic target for patients with ESCC.

## 1. Introduction

Esophageal cancer ranks as the ninth leading cause of cancer deaths in Taiwan, and more than 90% of esophageal cancer was esophageal squamous cell carcinoma (ESCC) [1]. Despite advances in the diagnosis and treatment of ESCC in recent decades, the prognosis of patients with ESCC still remains unsatisfactory [2,3,4]. The 5-year survival rate of patients diagnosed with ESCC is approximately 20~40% [4,5]. Therefore, the discovery of biomarkers for ESCC prognosis may improve risk-adapted treatment strategies and lead to the identification of a novel target for ESCC.

The ubiquitously transcribed TPR gene on the X chromosome (*UTX*), also known as KDM6A, has been identified as a histone demethylase that specifically targets di- and tri-methyl groups on lysine 27 of histone H3 (H3K27me2/3) and has been proven to be essential during cellular reprogramming [6], embryonic development, and tissue-specific differentiation [7]. In 2009, inactivated somatic mutations and deletions targeting the *UTX* gene were identified in a variety of human cancers including multiple myeloma, medulloblastoma, esophageal, colon, bladder, prostate, and renal cancer [8,9,10,11]. Constitutional inactivation of *UTX* causes a specific hereditary disorder called the Kabuki syndrome which may develop into several types of cancer such as neuroblastoma, hepatoblastoma, acute leukemia, and fibromyxoid sarcoma, suggesting that Kabuki syndrome is a cancer predisposition syndrome [12]. 

Kabuki individuals with mutations in *UTX* have been identified in both female and male patients [13]. Kabuki syndrome results from hypomorphic female heterozygous mutation and null male hemizygous mutation of *UTX* [14]. A recent study indicated that Kabuki causative *UTX* protein mutations vary from complete *UTX* deletion to single amino acid point substitutions. However, more precise molecular mechanisms of these *UTX* mutations in cells or mouse models should be further investigated.

In addition, *UTX* gene was identified as one of the 127 significantly mutated genes in The Cancer Genome Atlas (TCGA) study in which whole-exome sequencing was performed on 3281 tumors derived from 12 tumor types [15]. *UTX* was downregulated in multiple myeloma cell lines leading to an increase in cell growth [16]. Decreased *UTX* also induced the expression of adhesion factors, including *AOC3*, *CDHR5*, and *NCAM1* that are involved in cell reattachment upon dissemination. On the other hand, *UTX* was identified as a prooncogenic cofactor essential for leukemia maintenance in class II basic helix–loop–helix (bHLH) protein TAL1-positive (but not TAL1-negative) T-cell acute lymphoblastic leukemia [17]. Meanwhile, Kim et al. reported that *UTX* contributes to breast cancer cell proliferation with high levels of *UTX* being associated with poor prognosis in patients with breast cancer [18]. In cervical and head and neck tumors, HPV (human papillomavirus)-positive tumors were found to express higher levels of KDM6A [19]. These results indicated the complicated role of *UTX* in the pathogenesis of cancer. To the best of our knowledge, although *UTX* defects have been reported in ESCC [11], the prognostic significance of *UTX* expression in patients with ESCC remains largely undefined. Therefore, we conducted the present study to investigate this issue further.

## 2. Results

### 2.1. Patient Characteristics

A total of 106 patients with ESCC who had received surgery were considered in this study. The patients had a median age of 55 years (range, 29–80 years), and the characteristics of the patients are further summarized in Table 1. Among them, 101 (95%) were men and 5 (5%) were women. In terms of T classification, 42 (40%) of the patients were T1; 28 (26%) were T2; 26 (25%) were T3; and 10 (9%) were T4. Furthermore, in terms of N classification, 70 (66%) of the patients were N0; 25 (24%) were N1; 9 (8%) were N2; and 2 (2%) were N3. In terms of the 7th edition American Joint Committee on Cancer AJCC stages staging system 5 (5%) of the patients were stage IA, 17 (16%) were stage IIA; 26 (24%) were stage IIB; 11 (10%) were stage IIIA; 3 (3%) were stage IIIB; 9 (9%) were stage IIIC; and 2 (2%) were stage IV. Further analyses of histologic grades showed a grade 1 lesion in of the 10 (9%) patients, grade 2 in 70 (66%) of the patients, and grade 3 in 26 (25%) of the patients. Primary tumor location was found to be upper in 19 (18%) of the patients, middle in 36 (34%) of the patients, and lower in 51 (48%) of the patients. Among all 106 patients, resection margins were positive for residual tumor in 15 (14%) of the patients. At the time of analysis, the median periods of follow-up were 66 months (range, 61–112 months) for the 46 survivors and 53 months (range, 3.5–112 months) for all 106 patients. The 5-year survival (OS) and disease-free survival (DFS) of these 106 patients were 48% and 43%, respectively.

### 2.2. Correlation between Clinicopathologic Parameters and UTX Expression

**Figure 1:**
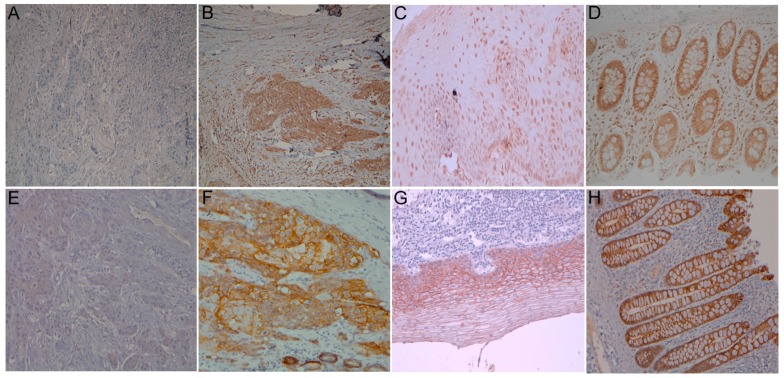
Immunohistochemical staining of *UTX* in esophageal squamous cell carcinoma (ESCC). (**A**) Representative example of low *UTX* expression. Original magnification, ×100; (**B**) Representative example of high *UTX* expression. Original magnification, ×100; (**C**) *UTX* immunoreactivity was present in adjacent normal esophageal mucosa. Original magnification, ×200; (**D**) *UTX* immunoreactivity was present in colon mucosa used as a positive control. Original magnification, ×200; (**E**) Representative example of low E-cadherin expression. Original magnification, ×200; (**F**) Representative example of high E-cadherin expression. Original magnification, ×200; (**G**) E-cadherin immunoreactivity was present in adjacent normal esophageal mucosa. Original magnification, ×200; (**H**) *UTX* immunoreactivity was present in colon mucosa used as a positive control. Original magnification, ×200.

Among the 106 patients considered, high *UTX* expression was identified in 44 (42%) of the patients (Figure 1). The associations between the clinicopathological parameters and *UTX* expression are summarized in Table 2. We did not observe any association between *UTX* expression and any clinicopathologic parameters including age, primary tumor location, histologic grading, T classification, N classification, and 7th edition American Joint Committee on Cancer (AJCC) Stage. 

### 2.3. Survival Analyses

**Figure 2:**
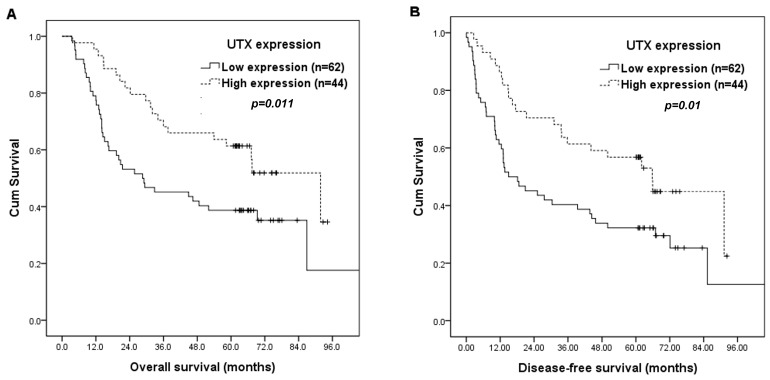
Kaplan-Meier curves according to *UTX* status. (**A**) OS rate according to *UTX* status; (**B**) DFS rate according to *UTX* status.

The correlations of patients’ survival with clinicopathological parameters and *UTX* expression are summarized in Table 3. By log-rank tests, 7th edition AJCC stage I (*p* = 0.008), T classification, T1/2 (*p* = 0.013), N classification, N0 (*p* < 0.001), negative surgical margin (*p* = 0.015), and high *UTX* expression (*p* = 0.011, Figure 2A) were associated with superior OS. Furthermore, 7th AJCC stage I (*p* = 0.002), T classification, T1/2 (*p* = 0.022), N classification, N0 (*p* < 0.001), and high *UTX* expression (*p* = 0.01, Figure 2B) were associated with better DFS. 

In multivariate analysis, high *UTX* expression (*p* = 0.013, hazard ratio = 1.996, 95% confidence interval: 1.160~3.436) remained independently associated with superior OS, together with N classification, N0 (*p* < 0.001, hazard ratio = 3.819, 95% confidence interval: 2.250~6.480). For DFS, high *UTX* expression (*p* = 0.009, hazard ratio = 1.972, 95% confidence interval: 1.189~3.279) and N classification (*p* < 0.001, hazard ratio = 3.350, 95% confidence interval: 2.040~5.502) represented an independent adverse prognosticator. The 5-year OS and DFS rates were 61% and 57% respectively, in patients with high *UTX* expression, and 39% and 32% respectively, in patients with low *UTX* expression.

Moreover, we further tested whether the combination of *UTX* and E-cadherin expression can improve the prognostic value. We found that the combination of low *UTX* and low E-cadherin expression robustly identifies the group of patients with extremely poor prognosis. The 5-year OS (*p* = 0.001) and DFS (*p* < 0.001) rates were 31% and 22%, respectively, in the 36 patients with low *UTX* and E-cadherin expression, and 57% and 53%, respectively, in the 70 patients without low *UTX* and E-cadherin expression.

### 2.4. Inhibition of UTX Promoted ESCC Cells Proliferation and Epithelial-Mesenchymal Transition Process

**Figure 3:**
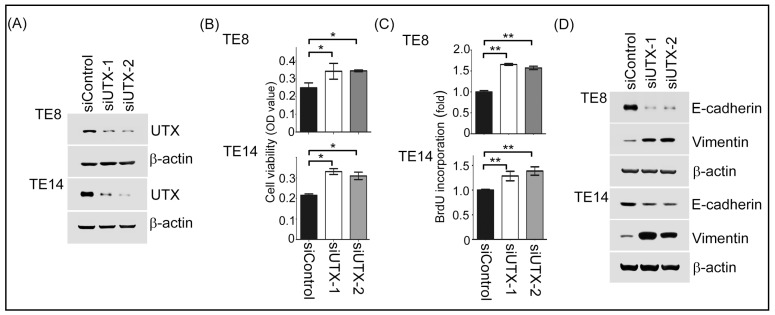
*UTX* expression regulated cell growth and epithelial-mesenchymal transition (EMT) expression in TE8 and TE14 cells. (**A**) The protein expression level of *UTX* was demonstrated in TE8 cells transfected with siControl and si*UTX* using Western blotting analysis; (**B**,**C**) The cell growth abilities of siControl and si*UTX* in TE8 and TE14 cells were measured by MTT (3-(4,5-dimethylthiazol-2-yl)-2,5-diphenyltetrazolium bromide) and BrdU assays; (**D**) The E-cadherin, and Vimentin protein expression levels were investigated with Western blotting analysis in TE8 and TE14 cells transfected with siControl and si*UTX*. *, *p* < 0.05; **, *p* < 0.01.

To study the functions of *UTX* in ESCC, we generated *UTX*-depleted ESCC cells in the TE8 and TE14 cell lines. The knockdown efficiency was revealed to be effective through Western blotting analysis (Figure 3A). Next, to examine if *UTX* modulated ESCC proliferation, we performed MTT (3-(4,5-dimethylthiazol-2-yl)-2,5-diphenyltetrazolium bromide) assay. As shown in Figure 3B, TE8 and TE14 cells transfected with *UTX*-mediated siRNA were grew faster than those transfected with siControl cells. Using the same cell panels, similar results were also observed with a bromodeoxyuridine BrdU incorporation assay (Figure 3C). Moreover, *UTX*-depleted TE8 and TE14 cells reduced the expressions of epithelial marker E-cadherin and increased those of Vimentin, a mesenchymal marker (Figure 3D). Together, these results reveal that *UTX* prevention may induce cell proliferation and participate in the epithelial-mesenchymal transition (EMT) process in ESCC cells. To evaluate the potential relevance of the above in vitro findings in ESCC in a clinical setting, we also analyzed the protein expressions of E-cadherin in tissues samples from the 106 human ESCC patients using immunohistochemistry. There was a significant correlation between *UTX* expression and E-cadherin expression (*p* = 0.008, Table 2). Representative staining results of E-cadherin expression are shown in Figure 1. 

## 3. Discussion

As is well known, epigenetic modifications, such as histone methylation or demethylation are involved in gene activation. *UTX*, a histone demethylase, removes di- and tri-methyl groups on histone H3 lysine 27 (H3K27) which is essential for tissue differentiation, cell maintenance, cell reprogramming and cancer development [20]. Using exome- or genome-wide sequencing strategies and TCGA databases, various somatic mutations and deletions of *UTX* have been found in several human cancers, with a prevalence of 24.24% in bladder cancer, 10% in prostate cancer, and 1.85% in esophageal cancer [20]. Interestingly, induction of *UTX* expression in *UTX*-depleted cells results in a K3K27me3 decrease and attenuates cell proliferation [11]. In addition, in normal fibroblast cells, *UTX* expression activates the Rb (Retinoblastoma) pathway to suppress cell growth, indicating that *UTX* may play the role of tumor suppressor in human cancers [21]. Conversely, in breast tumors, *UTX* is rarely mutated and its upregulation is correlated with poor prognosis [22]. *UTX* knockdown in leukemia cell lines exhibits an anti-growth effect [23]. These conflicting results imply that the function of *UTX* in human cancers may depend on cell-context manner. In the present study, we found that positive *UTX* expression was associated with better clinical outcomes in 106 patients with ESCC receiving esophagectomy and blockage of endogenous *UTX* by the siRNA approach led to an increase in cell proliferation and elevation of the BrdU incorporation ability in ESCC cells, suggesting that *UTX* acts a tumor suppressor in ESCC.

EMT plays a critical role in cancer progression. EMT allows cells to prevent death and promotes migratory and invasive abilities. An abundance of evidence indicates that an epigenetic regulator plays a vital role in controlling EMT and cancer progression. Several previous studies [24] have shown that reduced E-cadherin expression is a poor prognosticator in patients with ESCC. In our study, we found that decreased E-cadherin expression was associated with higher T classification, higher N classification, advanced 7th edition AJCC stages, and inferior OS rates in 106 ESCC patients receiving esophagectomy. With regard to colon cancer cells, Zha et al. [25] and Zhou et al. [8] reported the inactivation of *UTX* down-regulated E-cadherin gene expression. For breast cancer cells, Choi et al. [26] found that *UTX* loss induced EMT. However, the role of *UTX* in the regulation of EMT expression is still unclear in ESCC cells. Here, we demonstrated that loss-of function of *UTX* decreased E-cadherin expression and increased vimentin expression in ESCC cells, indicating that *UTX* might be involved in the regulation of the EMT process in ESCC.

Our study has one critical limitation in that it is a retrospective analysis and is based on a relatively small number of patients.

In conclusion, high *UTX* expression is independently associated with better prognosis in patients with ESCC. In ESCC cell lines, downregulation of *UTX* increases cell growth and decreases E-cadherin expression. Our results may further elucidate the role of *UTX* in ESCC and provide a potential therapeutic target for patients with ESCC.

## 4. Materials and Methods 

### 4.1. Patient Population

We retrospectively reviewed ESCC patients receiving esophagectomy at Kaohsiung Chang Gung Memorial Hospital. Approval to analyze and publish the aggregated anonymous data was given by the Institutional Review Board committee of Kaohsiung Chang Gung Memorial Hospital at Kaohsiung, Taiwan. The approval number of this project was 104-7233B. The approval date was on 17 November 2015. We excluded patients with synchronous cancers in other organs and patients receiving preoperative chemoradiotherapy, preoperative chemotherapy, or preoperative radiotherapy. Finally, 106 patients were identified. Patients undergoing surgery had a radical esophagectomy with cervical esophagogastric anastomosis (McKeown procedure) or an Ivor Lewis esophagectomy with intrathoracic anastomosis, reconstruction of the digestive tract with a gastric tube, and pylorus drainage procedures. All patients received two-field lymph node dissection. The pathological tumor node metastasis (TNM) staging system was determined according to the 7th edition AJCC staging system [27]. The OS rate was determined from the time of surgery to death as a result of all causes. The DFS rate was computed from the time of surgery to the recurrence of disease or death from any cause without evidence of recurrence. 

### 4.2. Immunohistochemistry 

Immunohistochemistry staining was performed using an immunoperoxidase technique. Staining was performed on slides (4 mm) of formalin-fixed, paraffin-embedded tissue sections with primary antibodies against *UTX* (Clone D3Q1l, 1:400, Cell Signaling Technology, Boston, MA, USA) and E-cadherin (BD610182, 1:2000, BD Biosciences, Sparks, MD,). Briefly, after deparaffinization and rehydration, the retrieval of the antigen was performed by treating the slides in 10 mmol/L citrate buffer (pH 6.0) in a hot water bath (95 °C) for 20 min. Endogenous peroxidase activity was blocked for 15 min in 0.3% hydrogen peroxide. After blocking with 1% goat serum for 1 h at room temperature, the sections were incubated with primary antibodies for at least 18 h at 4 °C overnight. Immunodetection was performed using the LSAB2 kit (Dako, Carpinteria, CA, USA) followed by 3-3′-diaminobenzidine for color development and hematoxylin for counterstaining. Incubation without the primary antibody was used as a negative control, and normal colon mucosa was used as a positive control for *UTX* and E-cadherin. The staining was assessed by 2 pathologists (Sheng-Lan Wang and Wan-Ting Huang without any information on clinicopathologic features or treatment outcome. A semi-quantitative immunoreactive score (IRS) was used to evaluate the immunohistochemistry staining. [28] The IRS was calculated by multiplying the staining intensity (graded as: 0 = no staining, 1 = weak staining, 2 = moderate staining, and 3 = strong staining) with the percentage of positively stained cells (0 = no stained cell, 1 = <10% of stained cells, 2 = 10–50% of stained cells, 3 = 51–80% of stained cells, and 4 = >80% of stained cells). The criterion for positive staining was a specimen with a IRS > 4. 

### 4.3. Cell Lines and siRNA Transfection

The human esophageal cell lines TE8 were obtained from the American Type Culture Collection, and maintained in Dulbecco’s Modified Eagle’s Medium (DMEM), supplemented with 10% fetal bovine serum at 37 °C and 5% CO_2_. For siRNA transfection experiments, two synthetic *UTX* targeting siRNAs and one sicontrol were applied. Cells were transfected with *UTX*-mediated siRNA (50 nM) in serum-free DMEM using Plus/Lipofetamin Transfection Reagent according to the manufacturer’s instructions. Two double-stranded synthetic RNA oligomers (5′-GAACAGCUCCGCGCAAAUA-3′ and 5′-GAGAGUAAUUCACGAAAGA-3′) deduced from human *UTX*, and one siRNA control (#4611G; Ambion, Austin, TX, USA) were used in the siRNA experiments.

### 4.4. Western Blotting Assay

TE8 and TE14 cells transfected with a negative control or *UTX*-siRNA were harvested and homogenized in RIPA (Radio-Immunoprecipitation Assay) lysis buffer, containing protease inhibitor cocktail on ice for 30 min. The homogenate was centrifuged at 4 °C at 15,000 rpm for 30 min, and lysates were collected. Protein concentration was determined using the BCA Protein Assay kit (Thermo Fisher, Waltham, MA, USA). The cell lysates were subjected to the 10% SDS-PAGE gels for following western blotting. The primary antibodies (anti-*UTX*, anti-E-cadherin, anti-Vimentin, and anti-β-actin) were obtained from Cell Signaling Technology (Danvers, MA, USA). Horseradish peroxidase (HRP)-conjugated secondary antibodies were obtained from Sigma (St. Louis, MO, USA). 

### 4.5. MTT and BrdU Assay

Cells were seeded at a density of 1 × 10^4^ cells per well in 96-well plates and maintained in DMEM supplemented with 10% FBS (fatal bovine serum). After overnight incubation, the culture medium was removed and the cells were washed with PBS (phosphate buffer saline). The freshly completed DMEM (Dulbecco’s Modified Eagle Medium) medium was added and cultured for 48 h. Following this that the culture medium was removed and the cells were washed with PBS. The cells were then incubated with 0.5 mg/mL MTT (3-(4,5-dimethylthiazol-2-yl)-2,5-diphenyltetrazolium bromide), in a culture medium without FBS, for 4 h at 37 °C in a 5% CO_2_ atmosphere. The medium was removed and 100 μL DMSO (Dimethyl sulfoxide) buffer was added and incubated in the dark for 10 min. Absorbance was measured on a microplate reader at 540 nm. For bromodeoxyuridine (BrdU) incorporation assay, cells were seeded at a density of 5 × 10^3^ cells/well in 96-well culture plates for 48 h. BrdU incorporation analysis was performed using a cell proliferation ELISA kit (Roche Diagnostics, Mannheim, Germany) according to the manufacturer’s instructions.

### 4.6. Statistical Analysis

For patient data, statistical analysis was performed using the SPSS 17 software package (SPSS Inc., Chicago, IL, USA). The chi-square test and Fisher’s exact test were used to compare data between the two groups. For the survival analysis, the Kaplan-Meier method was used for univariate analysis, and the difference between the survival curves was tested by a log-rank test. In a stepwise forward fashion, significant parameters at a univariate level were entered into a Cox regression model to analyze their relative prognostic value. For all analyses, two-sided tests of significance were used with *p* < 0.05 being considered significant.

## Figures and Tables

**Table 1:** Characteristics of 106 patients with esophageal squamous cell carcinoma (ESCC) receiving esophagectomy.

Age (years)	Parameters	No. of Cases (%)
	median	55
	mean	56
	range	29–80
**Sex**		
	male	101 (95%)
	female	5 (5%)
**Primary tumor location**		
	Upper	19 (18%)
	Middle	36 (34%)
	Lower	51 (48%)
**T classification**		
	T1	42 (40%)
	T2	28 (26%)
	T3	26 (25%)
	T4	10 (9%)
**N classification**		
	N0	70 (66%)
	N1	25 (24%)
	N2	9 (8%)
	N3	2 (2%)
**7th AJCC Stage**		
	IA	5 (5%)
	IB	33 (31%)
	IIA	17 (16%)
	IIB	26 (24%)
	IIIA	11 (10%)
	IIIB	3 (3%)
	IIIC	9 (9%)
	IV	2 (2%)
**Histological grading**		
	Grade 1	10 (9%)
	Grade 2	70 (66%)
	Grade 3	26 (25%)
**Surgical margin**		
	Negative	91 (86%)
	Positive	15 (14%)
***UTX*** **expression**		
	Low expression	62 (58%)
	High expression	44 (42%)
**E-cadherin**		
	Low expression	50 (47%)
	High expression	56 (53%)

AJCC, American Joint Committee on Cancer.

**Table 2:** Associations between *UTX* expression and clinicopathological parameters in 106 patients with ESCC receiving esophagectomy.

Parameters		*UTX* Expression			E-Cadherin Expression		
		Low	High	*p* Value	Low	High	*p* Value
Age (years)	<55	29	23	0.58	25	27	0.85
	≥55	33	21		25	29	
Primary tumor location	U + M	30	25	0.39	22	33	0.13
	L	32	19		28	23	
T classification	T1 + T2	38	32	0.22	26	44	0.004 *
	T3 + T4	24	12		24	12	
N classification	N0	37	33	0.10	25	45	0.001 *
	N1 + 2 + 3	25	11		25	11	
7th AJCC Stage	I	20	18	0.36	12	26	0.016 *
	II + III + IV	42	26		38	30	
Histological grading	Grade 1 + 2	44	36	0.20	34	46	0.091
	Grade 3	18	8		16	10	
Surgical margin	Negative	50	41	0.068	40	51	0.10
	Positive	12	3		10	5	
E-cadherin expression	Low	36	14	0.008 *	-	-	-
	High	26	30		-	-	-

AJCC, American Joint Committee on Cancer. * Statistically significant.

**Table 3:** Results of univariate log-rank analysis of prognostic factors for overall survival (OS) and disease-free survival (DFS) in 106 patients with ESCC receiving esophagectomy.

Factors	No. of Patients	Overall Survival (OS)		Disease-Free Survival (DFS)	
		5-year OS (%)	*p* Value	5-year DFS (%)	*p* Value
Age (years)					
<55	52	56%	0.32	52%	0.17
≥55	54	41%		33%	
Location					
U + M	55	55%	0.20	46%	0.42
L	51	41%		39%	
T classification					
T1 + 2	70	56%	0.013 *	49%	0.022 *
T3 + 4	36	33%		31%	
N classification					
N0	70	63%	<0.001 *	54%	<0.001 *
N1 + 2 + 3	36	19%		19%	
7th AJCC stage					
I	38	63%	0.008 *	58%	0.002 *
II + III + IV	68	40%		34%	
Histological grading					
Grade 1 + 2	80	50%	0.33	43%	0.62
Grade 3	26	42%		42%	
Surgical margin					
Negative	91	52%	0.015 *	45%	0.11
Positive	15	27%		27%	
*UTX* expression					
Low expression	62	39%	0.011 *	32%	0.01 *
High expression	44	61%		57%	
E-cadherin expression					
Low expression	50	38%	0.026 *	28%	0.005 *
High expression	56	57%		55%	
Low *UTX*/E-cadherin expression					
Presence	36	31%	0.001 *	22%	<0.001 *
Absence	70	57%		53%	

AJCC, American Joint Committee on Cancer. * Statistically significant.

## Abbreviations

*UTX*	ubiquitouslytranscribed TPR gene on the X chromosome
ESCC	Directory of open access journals
H3K27me2/3	lysine 27 of histone H3
TCGA	The Cancer Genome Atlas
bHLH	basic helix–loop–helix
DFS	Disease-free survival
